# The role of the anterior temporal lobes in the comprehension of concrete and abstract words: rTMS evidence

**DOI:** 10.1016/j.cortex.2009.02.006

**Published:** 2009-10

**Authors:** Gorana Pobric, Matthew A. Lambon Ralph, Elizabeth Jefferies

**Affiliations:** a Neuroscience and Aphasia Research Unit, School of Psychological Sciences, University of Manchester, UK; b Department of Psychology, University of York, UK

**Keywords:** Semantic cognition, Repetitive transcranial magnetic stimulation, Word imageability, Anterior temporal lobes

## Abstract

Conceptual knowledge allows us to bring meaning to our world. Studies of semantic dementia (SD) patients and some functional neuroimaging studies indicate that the anterior temporal lobes, bilaterally, are a core neural substrate for the formation of conceptual representations. The majority of SD patients (who have circumscribed atrophy of the anterior temporal lobes) have better comprehension of concrete than abstract words. However, this finding remains controversial, as some individual SD patients have exhibited reverse imageability effects, i.e., relative preservation of abstract knowledge. This would imply that the anterior temporal lobes are particularly crucial for processing sensory aspects of semantic knowledge, which are an important part of concrete but not abstract concepts. To adjudicate on this debate, we used offline, low-frequency, repetitive transcranial magnetic stimulation to disrupt neural processing temporarily in the left or right temporal poles (TPs). We examined this effect using a synonym judgement task, comprising high, medium and low imageability items, which we have previously employed with a case-series of SD patients. The time required to make semantic decisions was slowed considerably, particularly for low imageability items, consistent with the pattern we observed in SD. These results confirm that both TPs make a critical contribution to semantic processing, even for abstract concepts that do not have strong sensory representations.

## 1 Introduction

Semantic memory encompasses the meaning of all types of verbal and non-verbal stimuli including words, pictures, objects, environmental sounds and faces. It also allows us to express knowledge in a wide variety of domains, both verbal (e.g., naming and verbal definitions) and non-verbal (e.g., drawing and object use). Perhaps even more importantly, our semantic representations allow us to generalise knowledge appropriately from one exemplar to another (Lambon Ralph and Patterson, 2008). As such, semantic memory is integral to our everyday lives and semantic impairments are extremely debilitating. Therefore, the neural correlates of conceptual knowledge are a topic of fundamental interest in cognitive neuroscience.

At the present time, there is considerable debate in the literature about the putative roles of different brain regions in semantic cognition, with strong advocates for the importance of one brain region over another (Hickok and Poeppel, 2007; Martin, 2007; Patterson et al., 2007; Wise, 2003). An overview of neuropsychological and neuroimaging studies suggest that semantic cognition is supported by a three-part neural network made up of the left prefrontal cortex, the temporoparietal junction and the temporal poles (TPs) bilaterally (Jefferies and Lambon Ralph, 2006). Although there is convergent evidence for the involvement of the first two regions, the argument for the involvement of the TP rests heavily upon neuropsychological evidence from semantic dementia (SD) patients (Wise, 2003). Patients with SD have a highly specific impairment of semantic memory: they fail to diverse semantic tasks even though other aspects of cognition and language – such as phonology, visual processing and decision-making – remain intact (Hodges et al., 1992; Snowden et al., 1989). The selective nature of the semantic impairment is coupled with a specific pattern of brain damage: SD patients have bilateral atrophy and hypometabolism in the anterior temporal lobes, maximal in the inferior and lateral aspects, and the extent of this atrophy correlates with the severity of the semantic impairment (Mummery et al., 2000; Nestor et al., 2006). Whilst the brain damage in SD is remarkably circumscribed and consistent across patients, it is always possible that the semantic impairment actually results from pathology in regions beyond those maximally damaged. In addition, because SD is characterised by bilateral atrophy, it is not possible to investigate the roles of left and right anterior temporal lobe (ATL) in isolation. Therefore, the contributions of the ATL to semantic processing are not absolutely defined on the basis of this neuropsychological evidence alone.

Recently we used repetitive transcranial magnetic stimulation (rTMS) to disrupt processing within the ATL in normal volunteers (Pobric et al., 2007; Lambon Ralph et al., 2009). We demonstrated that the behavioural pattern in SD can be mirrored in neurologically intact participants. Temporary disruption to neural processing in the ATL produces a selective semantic impairment leading to significant slowing of both picture naming and word comprehension but not other equally demanding non-semantic cognitive tasks. The successful application of rTMS over the ATL region licenses the use of this technique to explore other key research questions about the nature semantic representations in the ATL.

An important topic concerns the representation and processing of the meanings of concrete and abstract words. Concrete concepts (e.g., glass) encapsulate the meanings of tangible things that can be experienced through our senses – consequently, we can readily form mental images for concrete words. Abstract concepts (e.g., happiness), in contrast, do not refer to physical objects and, for the most part, do not readily evoke mental images: instead these concepts refer to ideas or mental states. In behavioural studies, healthy participants often show faster and more accurate processing for imageable words (DeGroot, 1989; James, 1975; Kroll and Merves, 1986). Patients with brain damage normally show an exaggeration of this effect – for example, people with aphasia and deep dyslexia typically make many more errors for abstract than concrete items (Coltheart, 1980; Goodglass et al., 1969; Jefferies et al., 2007). Concrete items have sensory referents, whereas abstract items do not (Paivio, 1986). This might result in concrete items having more semantic features or richer semantic representations for these items (Jones, 1985; Plaut and Shallice, 1993), explaining the normal processing advantage for concrete over abstract concepts. However, a small number of patients with ATL damage in the context of SD or herpes simplex encephalitis have shown *reverse* imageability effects; i.e., relative preservation of abstract knowledge (Breedin et al., 1994; Cipolotti and Warrington, 1995; Reilly et al., 2006; Sirigu et al., 1991; Warrington, 1975; Yi et al., 2007). This led some groups to argue that reverse imageability effects are the norm in SD (Grossman and Ash, 2004). The double dissociation provided by these patients is important because it suggests that the cognitive and neural organisation of concrete and abstract concepts may be partially distinct: SD patients who show reverse concreteness effects might have damage to ATL areas that process sensory aspects of semantic knowledge. However, in a recent case-series study, we examined the comprehension of concrete and abstract concepts in twelve patients with SD (Jefferies et al., in press). In every case, comprehension was worse for abstract words, suggesting that reverse imageability effects are *not* widespread in SD. This lack of consistency between studies makes it crucial to seek convergent evidence for the role of ATL in concrete and abstract concepts.

Functional neuroimaging studies of neurologically intact participants point to considerable overlap in the network representing abstract/imageable words, although some differences have also been observed. Temporal lobe sites showing greater activation for concrete compared with abstract words have been found in left posterior infero-temporal cortex, medial ATL bilaterally and left inferior TP (Fiebach and Friederici, 2003; Noppeney and Price, 2002; Sabsevitz et al., 2005; Whatmough et al., 2004). In contrast, sites showing greater activation for abstract words occurred in left posterior superior temporal areas and in the superior parts of the TP bilaterally, as well as in left inferior frontal gyrus (Binder et al., 2005; Kiehl et al., 1999; Noppeney and Price, 2004; Perani et al., 1999; Sabsevitz et al., 2005; Whatmough et al., 2004). These patterns are broadly consistent with the proposal that concrete concepts are more reliant on occipital-temporal areas that underpin visual object recognition (Ungerleider and Mishkin, 1982), while abstract concepts depend more on brain regions responsible for language comprehension (e.g., Scott et al., 2000). However, the functional neuroimaging findings are rather inconsistent, with peak activations for both concrete and abstract concepts in ATL; consequently, they do not unequivocally predict reverse imageability effects following damage to ATL.

This review of the literature generates at least two hypotheses about the role of the ATL in concrete and abstract knowledge. (1) If ATL damage reliably produces reverse imageability effects, this area could comprise the anterior end of the ventral visual stream, responsible for recognising and extracting meaning from concrete objects but not abstract words. (2) Alternatively, if ATL damage impairs both concrete and abstract concepts (giving rise to the standard concrete > abstract effect in errors), this area might be an amodal semantic “hub” (Rogers et al., 2004) that makes a critical contribution to all types of concept, irrespective of imageability.

The purpose of the current study is to investigate the impact of rTMS on the neural organisation of abstract and concrete concepts in the left and right ATL. If semantic memory is supported by the ATL bilaterally, rTMS over either the left or right TP should result in slower decision times on a synonym judgement task but not on an equally demanding, non-semantic control task (number matching). Moreover, by comparing the effect of TPs rTMS on concrete and abstract concepts, we will establish if this area is differentially important for sensory aspects of semantic knowledge or whether it makes a critical contribution to knowledge of both concrete and abstract concepts.

## 2 Methods

### 2.1 Design

A 2 × 2 × 2 within-participant factorial design was used, with site (left *vs* right), task (synonym *vs* number judgement) and TMS (no stimulation *vs* TP stimulation) as the three factors. The study used the “virtual lesion” method in which the train of rTMS is delivered offline (without a concurrent behavioural task). Then behavioural performance is probed during the temporary refractory period and compared to performance on the same task outside this refractory window. To control for general arousal effects induced by TMS, half of the participants produced their “baseline”, no TMS data before rTMS was applied. The other half provided their baseline at least 30 min after the end of rTMS.

Jahanshahi and Rothwell (2000) distinguished between “control site” and “control task” TMS designs. If one is interested in testing the neuroanatomical specificity of a region then the “control site” method is most appropriate. Alternatively, if one is interested in the functions of a specific region (as we are) then the control task method is more helpful in that one can start to gauge which range of activities/function the target region is involved in. As noted above, we already know that semantic cognition is not uniquely localised to the ATL. Thus in designing our experiment, the focus was to probe the range of functions supported by the ATL by using the control task method in which performance on semantic tasks was compared to equally demanding, non-semantic processes.

### 2.2 Participants

Twelve right-handed participants took part in the experiment (7 females; mean age = 20.7 years, SD = 4.89, 8 of the participants were previously reported by Lambon Ralph et al., 2009). All were native English speakers and strongly right-handed, yielding a laterality quotient of at least +90 on the Edinburgh Handedness Inventory (Oldfield, 1971). They were free from any history of neurological disease or mental illness and not on any medication. All had normal or corrected-to-normal vision. The experiment was reviewed and approved by the local research ethics board. Participants were reimbursed for their participation.

### 2.3 Stimuli

The synonym judgement task was based on a neuropsychological assessment that we have developed to test verbal comprehension in SD and other aphasic patient groups (Jefferies et al., in press). The TMS experiment included two versions containing 72 trials each (144 in total). In each trial, a probe word (e.g., rogue) was presented at the top of the screen, with three choices underneath – the target (e.g., scoundrel) and two unrelated distractors (e.g., polka and gasket). The 144 trials were split evenly between three imageability bands [mean imageability of probe words = 275 (17.3), 452 (26.0) and 622 (14.0) respectively, on a scale of 100–700; Medical Research Council (MRC) Psycholinguistic Database; Coltheart, 1981]. The high, medium and low imageability words ranges did not overlap. Both the targets and distracters were matched to the probe word for imageability. The number task also contained 144 trials. The format was the same: a probe number was presented at the top of the screen and underneath three number choices were given. Participants were required to select the number closest in value to the probe.

### 2.4 Task and procedure

A PC running E-Prime software (Psychology Software Tools Inc., Pittsburgh, USA) presented the stimuli and recorded the responses. Participants performed two synonym and number judgement tasks per experimental session to measure baseline and TMS performance. This order was counterbalanced across stimulation sites. The experiment began with a practice block of 6 trials for each stimulus set. Experimental trials were presented in a random order in 2 blocks of 72 trials. A fixation point appeared on the screen to signal the start of each trial. Stimuli (words, numbers) were presented until response followed by a blank screen interval of 500 msec. Participants were asked to indicate their choice by pressing one of three designated keys on a keyboard. The tasks and stimulation site were counterbalanced across participants. Left and right stimulations were conducted on two separate sessions that were at least 3 weeks apart (from 3–7weeks).

### 2.5 TMS

A MagStim Rapid2 (Magstim Co., Whitland, UK) stimulator with 2 external boosters was used (maximum output approx. 2.2 Tesla). Magnetic stimulation was applied using a 70-mm figure-of-eight coil. The structural T1-weighted MRI scans were co-registered with the participant's scalp using MRIreg (www.mricro.com/mrireg.html). Immediately prior to the TMS session, scalp coordinates were measured using an Ascension minibird (www.ascension-tech.com) magnetic tracking system. From the tip of the TP, we measured 10 mm posterior along the middle temporal gyrus. This point was used in each participant as an anatomical landmark for the TP. The location of the TP was identified on each participant and the scalp location directly above this site was marked. The left Montreal Neurological Institute (MNI) coordinates for the TP in standard space were (−53, 4, −32). The right TP corresponded to average MNI coordinates of (52, 2, −28) in standard space.

### 2.6 Stimulation parameters

Individual motor threshold was determined for every participant; stimulation was delivered to the optimal scalp position, from which the minimal intensity required to induce contraction of the relaxed contralateral abductor pollicis brevis muscle was established. Motor thresholds ranged from 41 to 65% of maximum stimulator output. Stimulation was delivered at 120% of motor threshold (average = 64% of maximum output). Participants received 10 min TMS active stimulation (1 Hz for 600 sec.) over to the TP. The coil was securely held against the left/right temple, centred over the site to be stimulated. This TMS protocol has been shown to produce behavioural effects that last for several minutes after stimulation (Hilgetag et al., 2001; Kosslyn et al., 1999).

### 2.7 Methodological considerations

An advantage of low-frequency rTMS is that the stimulation modulates the level of excitability of a given cortical area beyond the duration of the rTMS train itself (Knecht et al., 2002; Pascual-Leone et al., 1998). In the present design, behaviour was evaluated before and after rTMS. Therefore, a nonspecific disruption of performance due to discomfort, noise, muscle twitches and intersensory facilitation associated with rTMS during the task was avoided. Particular care was taken in the placing of the TP coil because TMS here is more unpleasant than over occipital or parietal areas. We manipulated coil orientation to find an orientation that minimised uncomfortable contractions of facial/neck muscles. The stimulation was tolerated well by all participants who come from a dedicated subject pool, pre-screened on their ability to tolerate this type of stimulation.

## 3 Results

### 3.1 Overall analyses

**Fig. 1:**
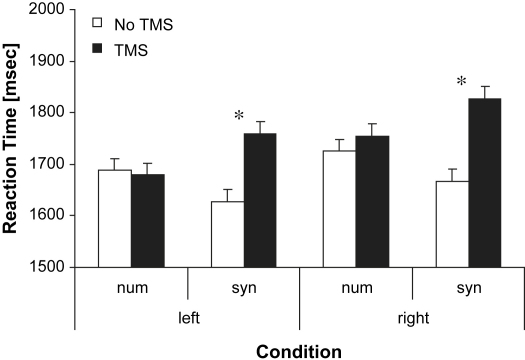
The effect of left or right TP stimulation on semantic and number judgement times. Each bar represents the mean decision time alongside the corresponding standard error adjusted for within subject comparisons (Loftus and Masson, 1994) for each condition. Syn = synonym judgement. Num = non-semantic number control task. Left = TMS over left TP. Right = TMS over right TP.

The participants' performance on the semantic task (timed synonym judgement) and the control task (timed number judgement) was compared with and without 10 min of offline 1 Hz rTMS over the left and right TP. Reaction times (RT) for all participants and conditions were examined in an analysis of variance (ANOVA) with task (synonym *vs* number judgement), site (left *vs* right TP) and TMS (rTMS *vs* no TMS) as within-subjects factors. There was no significant main effect of either task (*F* < 1, df = 1,11) or site (*F* < 1, df = 1,11); however, we observed a main effect of TMS (*F* = 27.05, df = 1,11, *p* < .001). There was a significant interaction between task and TMS (*F* = 14.88, df = 1,11, *p* = .002). Paired *t*-tests revealed that synonym judgement performance was significantly impaired by stimulation of both left TP [*t* (11) = 7.74, *p* < .001] and right TP [*t* (11) = 4.72, *p* < .001]. None of the *t*-tests for the number task were significant. The effects were carried in speed rather than accuracy. There was an overall effect of task on errors [number = 5.8% *vs* synonym judgement = 3.6%: *F* (1,11) = 9.02, *p* < .05] but there were no interactions with TMS or site Fig. 1.

### 3.2 Imageability analyses

**Fig. 2:**
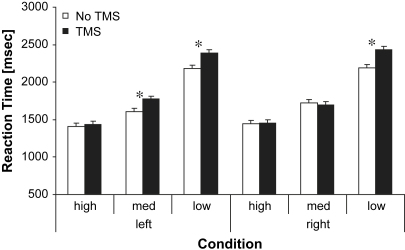
The TMS effect for high, medium and low imageability trials in the synonym judgement task. Each bar represents the mean decision time alongside the corresponding standard error adjusted for within subject comparisons (Loftus and Masson, 1994) for each condition. High = high imageability words. Med = medium imageability words. Low = low imageability words. Left = TMS over left TP. Right = TMS over right TP.

We examined RT for abstract versus concrete items in repeated-measures ANOVA with three within-subjects factors: site (left *vs* right TP), imageability (high, medium and low) and TMS (rTMS *vs* no TMS). There were significant main effects of imageability (*F* = 299.38, df = 2,22, *p* < .001) and TMS (*F* = 18.15, df = 1,11, *p* < .001). There was also a significant interaction between imageability and TMS (*F* = 6.86, df = 2,22, *p* < .05), which reflected a greater TMS effect for lower imageability items. Paired *t*-tests revealed that stimulation of left TP significantly impaired performance for medium imageability [*t* (11) = 2.37, *p* = .04] and low imageability items [*t* (11) = 2.71, *p* = .02]. Right TP stimulation also impaired processing of low imageability items [*t* (11) = 3.55, *p* = .004] Fig. 2.

### 3.3 Imageability error analyses

**Fig. 3:**
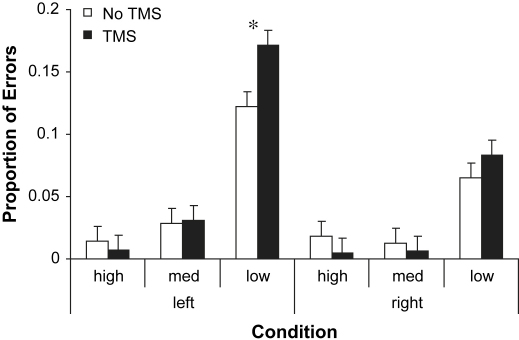
The proportion of errors induced by rTMS for each imageability condition in the synonym judgement task. Each bar represents the mean proportion of errors alongside the corresponding standard error adjusted for within subject comparisons (Loftus and Masson, 1994) for each condition. High = high imageability words. Med = medium imageability words. Low = low imageability words. Left = TMS over left TP. Right = TMS over right TP.

The error proportions for all participants and all conditions were examined in repeated-measures ANOVA with site (left *vs* right TP), imageability (high, medium and low) and TMS (rTMS *vs* no TMS) as factors. There was a main effect of imageability (*F* = 44.69, df = 2,22, *p* < .001) and a significant interaction between imageability and TMS (*F* = 3.99, df = 2,22, *p* < .05). Paired *t*-tests revealed that stimulation of left TP significantly increased the proportion of errors for low imageability items [*t* (11)=2.76, *p* < .05] but not high/medium imageability items. None of the *t*-tests for right TP were significant Fig. 3 .

## 4 General discussion

In this study we used rTMS to induce a “virtual lesion” or temporary slowing of processing in the left and right TP. We found that stimulation of both of these sites increased RT on a semantic task (synonym judgement) but not a control task matched for difficulty (number judgement), indicating that left and right TP make a critical contribution to semantic processing. In mathematical cognition, tasks requiring number magnitude judgements are regarded as semantic (Piazza et al., 2007). However, it has been shown that the neural basis of numerical concepts is independent of language (Gelman and Butterworth, 2005). These findings fit with neuropsychological studies of patients with SD and confirm the conclusions of our recent rTMS study with a larger sample size (Lambon Ralph et al., 2009).

For the first time, we also compared the impact of rTMS on the comprehension of concrete and abstract words. There was an interaction between TMS and imageability, reflecting more substantial effects of stimulation for abstract items. Participants were slower to process low/medium but not high imageability items following rTMS to both left and right TP. In addition, there were more errors for low imageability items following left-sided rTMS. Processing the meaning of abstract stimuli might require additional work within the ATL semantic system because these items are thought to be are less richly represented than concrete entities (Jones, 1985; Plaut and Shallice, 1993). This proposal is consistent with neuroimaging studies that have found greater TP activation for abstract than concrete items (e.g., Noppeney and Price, 2004).

Importantly, our findings are incompatible with the proposal that the TP (in either hemisphere) are differentially involved in visual/sensory aspects of semantic knowledge. This hypothesis predicts the opposite of the findings that we obtained. Although studies of individual patients with ATL damage (in the context of SD or herpes simplex encephalitis) have sometimes shown reverse imageability effects in comprehension, it appears that disruption of ATL processing does not *reliably* cause this effect. Instead, the current findings are consistent with a recent case-series study of SD employing the same synonym judgement task as this investigation (Jefferies et al., in press). Every patient in this study showed better comprehension of high than low imageability words, suggesting that although reverse imageability effects undeniably do occur in some individuals with SD, they are rare. SD patients who show this pattern might have an unusual distribution of atrophy (possibly focussed on medial or inferior posterior temporal regions, rather than the inferolateral TP). In addition, individual differences in educational level or premorbid experience might contribute to variability in the effect of imageability in SD. At least some of the patients who have previously shown reverse imageability have been highly educated professionals (e.g., Breedin et al., 1994; Warrington, 1975).

Our TMS findings indicate that both left and right ATL make a critical contribution to the processing of both concrete and abstract concepts. Although in this study a significant TMS effect was only observed for medium and low imageability items, we have previously demonstrated an rTMS effect for picture naming in the same left TP site (by definition, this task taps concrete knowledge; Pobric et al., 2007). These findings fit the notion of a single amodal semantic hub, represented bilaterally in left and right ATL (Rogers and McClelland, 2004). According to this view, ATL extracts amodal semantic knowledge from a distillation of information available in different input and output codes. From a neuroanatomical perspective, the ATL are an ideal substrate for forming amodal semantic representations as they are highly connected with other areas of modality-specific association cortex (Gloor, 1997). This idea has been implemented in a computational model incorporating a central semantic “hub” that receives inputs from both verbal and visual systems (Rogers et al., 2004). Units within this “hub” allow the model to extract high-order, amodal representations about concepts that are not dominated by similarities in any individual modality, but instead reflect semantic relationships apparent across all of the modality-specific representations taken together. These amodal semantic representations support the translation of information between different sensory and verbal modalities and promote correct semantic generalizations across items (Lambon Ralph and Patterson, 2008).

In sum, the results from the present rTMS study confirm that both TPs make a critical contribution to semantic processing, even for abstract concepts that do not have strong sensory representations. Future studies utilising rTMS will be able to explore whether more specific regions within the ATL are responsible for different aspects of imageability as indicated by some functional neuroimaging studies.
